# S164. “AT-RISK MENTAL STATES” PROGRAM IN LAUSANNE: INFLUENCE OF RECRUITMENT STRATEGIES ON THE RATE OF FALSE POSITIVES

**DOI:** 10.1093/schbul/sby018.951

**Published:** 2018-04-01

**Authors:** Alessandra Solida, Martine Cleusix, Carline Zorzi, Carina Ferrari, Kim Q Do, Philippe Conus

**Affiliations:** Lausanne University Hospital (CHUV)- CH

## Abstract

**Background:**

Various strategies have been proposed to improve recruitment of “at risk mental state” patients; they may have an impact on the type of patients who reach such programs. We describe the clinical program for “at-risk” patients implemented in 2014 in Lausanne and the characteristics of referrals over the years.

**Methods:**

Help seeking patients aged 14 to 35 were initially referred by health care providers for a specialized evaluation in case of suspicion of a potential “prodromal psychotic state” and more recently selected by PQ-16 (Ising et al. 2012) (cut-off: 6/16).

At-Risk Mental State (ARMS) was defined according to the Basic Symptoms criterion (COPER-COGDIS criteria) from the Schizophrenia Proneness Instrument – Adult version (SPI-A) and to the Clinical High Risk criteria of the Structured Interview for Prodromal Syndromes (SIPS). ARMS patients underwent an extensive clinical evaluation (including Mini-SCID, SOFAS, MARDS, Yung Mania Scale, etc.) and were followed-up every 6 months over 3 years.

**Results:**

Within a catchment area of 260 000 inhabitants, 110 patients have been referred to our center since 2014 and 100 completed the investigation. 29 (29%) fulfilled ARMS criteria, 52 (52%) didn’t and 19 (19%) were already psychotic.

The proportion of true ARMS patients decreased progressively over the years from 45% in 2014 and 2015, to only 22 and 13.9 % in 2016 and 2017.

In our sample of help-seekers, the group of patients ARMS- negative received mostly a schizophrenia spectrum diagnosis (26/52 patients, 50%), associated with low psychosocial functioning, even when not in the precise range of at-risk criteria.

**Discussion:**

The global prevalence (29%) of ARMS patients in our sample over the 4 years is marginally lower than previous reports on similar tertiary centers, which ranges from 33 to 51 % (Kline E., 2014). Our lower prevalence of ARMS patients within the sample may be linked to the limited resources we had to conduct an information strategy and our focus on psychologists and psychiatrists working at our department. The introduction in 2016 of more intense screening strategy based on the use of the PQ-16 lead to an increase in referral numbers but decreased the rate of ARMS among referred patients.

Our results confirm the influence of the recruitment strategy and information campaigns on the prevalence of at-risk patients within a population of help-seekers. The prevalence of schizophrenia spectrum diagnosis in our group of patients ARMS-negative also suggests that a larger “vulnerability” model for psychosis, more sensitive to functioning and negative symptoms and not narrowed on the focus of the risk of imminent acute psychosis, may better fit patients’ needs.

